# Quantifying the burden of lipid anomalies among adolescents in India

**DOI:** 10.1186/s12872-022-02819-y

**Published:** 2022-08-26

**Authors:** Kirti Kirti, Shri Kant Singh

**Affiliations:** Department of Survey Research and Data Analytics, International Institute for Population Sciences, Mumbai, Maharashtra India

**Keywords:** Lipid profile, Lipid anomalies, Hypercholesterolemia, Hypertriglyceridemia, HDL, LDL, Obesity, Diabetes, Micronutrients, Unhealthy diet, Adolescents, India

## Abstract

**Background:**

The present study’s aim is to quantify the burden of lipid abnormalities (excessive non-high-density lipoprotein (non-HDL) cholesterol and low-density lipoprotein (LDL) cholesterol) among Indian adolescents. Which has emerged as a significant covariate of coronary heart disease (CHD).

**Methods:**

The present study aims to unearth the prevalence of any lipid anomalies, their level, and types of lipid profiles among adolescents in India using the Comprehensive National Nutrition Survey 2016–18 i.e., cross-sectional data. Descriptive and bivariate statistical analyses have been used to check the associations and significant differences between groups of individuals suffering from any type of lipid abnormalities.

**Results:**

A total of 35,830 adolescents aged between 10 and 19 years (mean age:14.36 yrs.; SD = 2.81 for males and 14.39 yrs.; SD = 2.78 for females) were included. Roughly 77 percent of the adolescents are suffering from any lipid anomalies. Their mean lipid levels are 140.6 (SD = 32.9), 84.1 (SD = 24.8), 47.3 (SD = 10.7), and 95.3 (SD = 50.0) for total cholesterol, LDL, HDL, and triglycerides, respectively. A higher proportion of adolescents suffered from lipid anomalies among those who were overweight or obese (89%, 95% CI 85, 92) and pre-diabetics (81%, 95% CI 78, 83) compared to each of their counterparts. Furthermore, a considerable proportion of samples with vitamin A (70%, 95% CI 68, 73), D (81%, 95% CI 79, 82), and B12 deficits (73%,95% CI 72, 75), as well as zinc (77%, 95% CI 76, 77), folate (76%, 95% CI 74, 77), and iron deficits (75%,95% CI 73, 77), were suffering from any lipid anomalies. Of individuals who consume an unhealthy diet, 77% (95% CI 76, 78) of them were suffering from any lipid anomalies than others.

**Conclusions:**

The study contends that preventing the increasing burden of lipid abnormalities among Indian adolescents is essential. Vitamin and mineral deficiencies and unhealthy dietary habits are significantly associated with high LDL and non-HDL levels. In the longer run, this might cause the early onset of hypertension, diabetes, and CHDs. Hence, appropriate interventions are needed to curtail these early onsets by primarily focusing on adolescents.

## Background

Lipid abnormalities are when an individual’s blood contains excessive non-high-density lipoprotein (non-HDL) cholesterol and low-density lipoprotein (LDL) cholesterol, which has emerged as a significant factor of coronary heart disease (CHD) [1]. Cardiovascular diseases (CVDs) are majorly caused by abnormal lipid parameters or excessive non-high-density lipoprotein (non-HDL) cholesterol and low-density lipoprotein (LDL) cholesterol, known as hypercholesterolemia and adiposity [2, 3]. These are two primary components of metabolic syndrome [4]. Although hypercholesterolemia is less common in adolescents, yet, if present, then highly likely to increase the risk of CVDs at later ages. Hypercholesterolemia’s important secondary causes include diabetes [5]. Potential reasons for the increase in CVD rates include lifestyle changes associated with urbanization and epidemiologic and nutritional transitions accompanying economic development [6–8]. Dyslipidaemia has been closely linked to the pathophysiology of CVD and is a crucial independent modifiable risk factor for CVD [9]. While Asian Indians are known to have a unique pattern of dyslipidemia with lower HDL cholesterol, increased triglyceride levels, and higher proportions of small dense LDL cholesterol, there have been no large-scale representative studies on dyslipidemia to assess the magnitude of the problem in India [10].

Studies from the developed world have established that obesity among adolescents is associated with unfavourable lipid profiles [9]. Although in the Indian scenario, very few studies or perhaps no recent studies have been done to ascertain the situation among the younger generation [11].

Among the Indian population, a study has reported that one in every six individuals suffers from hypercholesterolemia [12]. Indian suburban children and adolescents aged 14 to 18 have reported notably low HDL levels paired with high BMI [13]. However, the same study reported that 23 percent of the population had total cholesterol greater than 170 mg/dl, 10 percent had LDL > 110 mg/dl, and 18 percent had triglyceride > 130 mg/dl [13]. Hence, it can be inferred that a palpable burden of lipid anomalies among adolescents is prevailing in India. Further, developing countries can be seen burdened with various adverse metabolic changes including lipid abnormalities [14, 15]. Consequently, leading to CVDs, diabetes, increased blood pressure, and cancer [14, 15]. Prevailing gender-based differences in lipid profiles can be seen. Females had significantly higher total cholesterol, HDL, and non-HDL cholesterol levels with a substantially lower LDL level than their counterparts [12, 13].

Hypercholesterolemia can be either due to a primary cause, genetic or familial, or secondary, acquired causes [1]. However, age is a vital risk factor for high cholesterol irrespective of gender [16–18]. High cholesterol can go undetected among young people aged 18–25 years [19]. Obesity, diabetes, and hypertension were strongly associated with dyslipidaemia [12].

There is a void between the general knowledge of lipid anomalies among adolescents in India. As a result, the current study is necessary. Studies to date are based on sub-regional and smaller sub-groups of India’s population, mainly deriving data from the healthcare sources [10, 11, 13, 20]. Also, the studies based on nationally representative data include only specific section of the population [12]. Therefore, the current study assesses all types, levels, and patterns of lipid abnormalities, at the national level among children and adolescents aged 10 to 19 years. Along with lipid anomaly exploration, potential causes will also be analysed. It is crucial to assess and investigate the situation early on in this exceptional situation to prevent serious health risks at a later age. Researching cholesterol levels in children and adolescents is crucial since they are more likely to conduce unhealthy lifestyles and consume packaged and processed foods which are high in saturated fats and sugar which ultimately alters the lipid profile of an individual [17]. An early detection and intervention could avert negative consequences.

## Methods

The present study utilises secondary data from the “Comprehensive National Nutrition Survey (CNNS) (2016–18)” under the supervision of the Ministry of Health and Family Welfare, Government of India, in collaboration with UNICEF, conducted by the Population Council. CNNS is India’s first-ever nationally representative nutrition survey of children and adolescents. In addition, the survey collected data on lipid profiles among individuals aged 5–19 years. The data can be obtained by reasonable request to the population Council website: https://www.popcouncil.org/uploads/pdfs/2019RH_CNNSreport.pdf.

### Study population

However, the study population for the present research will include adolescents only. As per WHO definition, the age range for adolescents is between 10 to 19 years [21]. Hence, a sample of 35,830 adolescents (i.e., the whole sample that CNNS has for the age range 10–19 years) with 17,865 males and 17,965 female adolescents, to evaluate the lipid profiles using viable descriptive statistics and tests.

### Derivation of measures used in the study

CNNS collected the Lipid profiles among children and adolescents aged 5 to 19. However, children and adolescents aged 10–19 years have been included in the study. Lipid profile primarily includes total serum cholesterol (assessed by spectrophotometry using cholesterol oxidase esterase peroxidase), high-density lipoprotein cholesterol (HDL-C) (assessed by spectrophotometry and direct measure polyethylene glycol-modified cholesterol oxidase), low-density lipoprotein cholesterol (LDL-C) (assessed by spectrophotometry and direct measure cholesterol oxidase), and triglycerides (assessed by spectrophotometry and enzymatic end point method).

### Operational definitions

Table 1 represents the cut-offs used for lipid profiles among adolescents were as per “National Cholesterol Education Programme (Expert Panel on Cholesterol Levels in Children, 2012)”[22].

**Table 1:** Cut-offs used for lipid profiles among adolescents

Lipid profile	Cut-offs for borderline/high (/low for HDL-C)
High total cholesterol	Serum cholesterol > 129 mg/dl
High triglycerides	Serum triglycerides > 129 mg/dl
Low HDL-C	Serum HDL-C < 40 mg/dl
High LDL-C	Serum direct LDL-C > 129 mg/dl
High total cholesterol: HDL-C ratio	Serum cholesterol to HDL-C > 4.5
Isolated high hypercholesterolemia	Serum cholesterol > 129 mg/dl and serum triglycerides < 129 mg/dl
Isolated high hypertriglyceridemia	Serum triglycerides > 129 mg/dl and serum cholesterol < 129 mg/dl
Isolated Low HDL-C	Serum HDL-C < 40 mg/dl without hypercholesterolemia or hypertriglyceridemia

Further, the outcome variable, whether an individual is suffering from any lipid anomalies, or no lipid anomalies, were derived from the combination of whether an individual is suffering from high total cholesterol, high LDL-C, high triglycerides, or low HDL-C. Therefore, they are categorized into two groups “0”: no lipid anomalies and “1”: any lipid anomalies.

Various background characteristics, including individual, anthropometric, and other household characteristics, were considered in the study by which the prevalence of any lipid anomalies was computed. Individual and household characteristics include age, gender, place of residence, wealth index, caste, region, and religion. Continuous anthropometric variables include triceps skinfold thickness (TSFT), mid-upper arm circumference (MUAC), and waist circumference (WC). Further, body mass index (BMI) is categorized into “0”: under or normal weight adolescents and “1” overweight or obese adolescents. Micronutrient deficiencies include vitamin A, D, and B12, and minerals include zinc, folate, and iron deficiencies.

Further derivation of the unhealthy diet index has been done using the MCA method using variables storing data on how frequently they consume fats/oils, sugar/jaggery, fried, junk foods, sweets, and aerated drinks. Finally, the index was divided into two categories “0”: normal/healthy diet index and “1”: unhealthy diet index.

### Data analyses

Firstly, descriptive analysis has been done to analyze the sample distribution across various background characteristics. In addition, a kernel density plot has been plotted for the standardized lipid profiles to examine the normality of the data. Further, a t-test has been used to test whether there is any statistical difference in mean lipid profiles by age and gender of the adolescents. Boxplot for lipid profiles has been plotted to address and compare the summary statistics by adolescent’s age and gender.

Further, bivariate analysis has been used to understand the prevalence of any lipid anomalies among adolescents aged 10–19 years by socioeconomic, demographic, and biological variables across India. National weights of biomarker has been used while exploring and computational analysis. STATA(SE) version 16.0 software has been used for data analysis and data wrangling.

## Results

Table 2 depicts the general characteristics of the adolescents aged 10 to 19 years based on the presence of any lipid anomalies by various individual and background characteristics in India. Adolescents who have any lipid anomalies have comparatively higher mean triceps skinfold thickness (TSFT) (Mean = 10.1 mm; SD = 4.9), mid-upper arm circumference (MUAC) (Mean = 22.0 cm SD = 3.1)), and waist-circumference (Mean = 64.1 cm; SD = 9.4) than those with no lipid anomalies.

**Table 2:** General characteristics of the adolescents aged 10 to 19 years based on the presence or absence of lipid anomalies in India, CNNS, 2018–20

Continuous variables	Total frequency (proportion)	No lipid anomalies	Any lipid anomalies
		Mean (SD)	
TSFT (in mm)	12,995 (100)	8.84 (4.20)	10.00 (4.90)
MUAC (in cm)	12,923 (100)	21.54 (3.24)	22.03 (3.70)
Waist-circumference (in cm)	12,921 (100)	62.72 (7.62)	64.08 (9.39)

Categorical variables		No lipid abnormality	Any lipid Abnormality
		Proportions (95% CI)	
**Age**			
Early Adolescents (10–14 years)	18,388 (51.85)	24.42 (23.42, 25.45)	75.58 (74.55, 76.58)
Late Adolescents (15–19 years)	17,442 (48.15)	22.34 (21.32, 23.40)	77.66 (76.60, 78.68)
**Gender**			
Male	18,425 (49.86)	25.24 (24.21, 26.31)	74.76 (73.69, 75.79)
Female	17,405 (50.14)	21.59 (20.6, 22.61)	78.41 (77.39, 79.40)
**Place of residence**			
Urban	19,606 (54.72)	24.13 (22.69, 25.62)	75.87 (74.38, 77.31)
Rural	16,224 (45.28)	23.20 (22.38, 24.05)	76.80 (75.95, 77.62)
**Wealth index**			
Poorest	3053 (20.01)	23.31 (21.71, 24.99)	76.69 (75.01, 78.29)
Poor	4688 (19.99)	23.10 (21.48, 24.81)	76.90 (75.19, 78.52)
Middle	6747 (20.01)	20.88 (19.39, 22.46)	79.12 (77.54, 80.61)
Rich	9053 (19.99)	25.29 (23.71, 26.93)	74.71 (73.07, 76.29)
Richest	12,289 (20.00)	24.56 (22.92, 26.28)	75.44 (73.72, 77.08)
**Caste**			
SC/STs	13,610 (33.39)	25.15 (23.87, 26.47)	74.85 (73.53, 76.13)
OBCs	11,419 (41.70)	23.26 (22.14, 24.41)	76.74 (75.59, 77.86)
Others	10,801 (24.90)	21.56 (20.21, 22.98)	78.44 (77.02, 79.79)
**Region**			
North	8217 (13.67)	25.74 (23.75, 27.84)	74.26 (72.16, 76.25)
Central	4020 (32.66)	25.52 (24.22, 26.87)	74.48 (73.13, 75.78)
East	5207 (23.44)	21.79 (20.39, 23.25)	78.21 (76.75, 79.61)
North-East	3954 (11.67)	23.03 (20.86, 25.35)	76.97 (74.65, 79.14)
West	5717 (15.52)	19.19 (17.58, 20.91)	80.81 (79.09, 82.42)
South	8715 (3.03)	28.92 (24.73, 33.50)	71.08 (66.50, 75.27)
**Religion**			
Hindu	24,916 (80.24)	24.30 (23.49, 25.12)	75.70 (74.88, 76.51)
Muslim	4629 (15.22)	19.56 (17.82, 21.43)	80.44 (78.57, 82.18)
Others	6285 (4.54)	20.18 (17.12, 23.64)	79.82 (76.36, 82.88)
**Mother's age**			
Less than 19	103 (0.29)	26.41 (13.00, 46.29)	73.59 (53.71, 87.00)
19–29 Years	1,571 (4.38)	23.21 (19.91, 26.87)	76.79 (73.13, 80.09)
30–39 Years	17,173 (47.93)	22.78 (21.77, 23.81)	77.22 (76.19, 78.23)
40–49 Years	12,178 (33.99)	24.37 (23.07, 25.72)	75.63 (74.28, 76.93)
50 and above	4805 (13.41)	23.76 (21.90, 25.74)	76.24 (74.26, 78.10)
**BMI**			
Under/normal weight	32,820 (95.89)	23.89 (23.15, 24.65)	76.11 (75.35, 76.85)
Overweight/obese	1408 (4.11)	10.93 (7.97, 14.82)	89.07 (85.18, 92.03)
Vitamin A deficit	1240 (13.50)	29.91 (27.51, 32.41)	70.09 (67.59, 72.49)
Vitamin D deficit	3959 (30.30)	19.34 (17.93, 20.84)	80.66 (79.16, 82.07)
Vitamin B12 deficit	2985 (25.32)	26.59 (25.13, 28.10)	73.41 (71.90, 74.87)
Zinc deficit	4202 (35.16)	23.31 (21.89, 24.79)	76.69 (75.68, 77.43)
Folate deficit	5923 (40.40)	24.38 (23.10, 25.72)	75.62 (74.28, 76.90)
Iron deficit	2499 (22.84)	25.11 (23.34, 26.97)	74.89 (73.03, 76.66)
**Prediabetes**			
No	10,663 (86.56)	23.43 (22.57, 24.32)	76.57 (75.68, 77.43)
Yes	1655 (13.44)	19.44 (17.15, 21.94)	80.56 (78.06, 82.85)
**Hypertension status**			
Normal	8947 (95.77)	22.90 (21.94, 23.88)	77.10 (76.12, 25.36)
Hypertensives	395 (4.23)	21.06 (17.32, 25.36)	78.94 (74.67, 82.68)
**Unhealthy diet index**			
Normal/Healthy	17,584 (49.08)	23.80 (22.79, 24.84)	76.20 (75.16, 77.21)
Unhealthy	18,240 (50.92)	23.06 (22.04, 24.11)	76.94 (75.89, 77.96)
**Iron folic supplements**			
Do not consume	32,177 (90.23)	23.64 (22.88, 24.42)	76.36 (75.58, 77.12)
Consume	3485 (9.77)	21.83 (19.54, 24.30)	78.17 (75.70, 80.46)
**Multivitamin supplements**			
Do not consume	31,805 (89.11)	23.60 (22.85, 24.37)	76.40 (75.63, 77.15)
Consume	3885 (10.89)	22.36 (19.96, 24.95)	77.64 (75.05, 80.04)
Total	35,830 (100)	23.44 (22.72, 24.17)	76.56 (75.83, 77.28)

Adolescents in the age-group 15–19 (i.e., late adolescents) years have higher proportions of the sample suffering from any lipid anomalies (i.e., 77.7%; 95% CI 76.6, 78.7) compared to those in early adolescent ages (i.e., 75.6%; 95% CI 74.6, 76.6). Female adolescents have a higher proportion with 78.4% (95% CI 77.4, 79.4) than male adolescents (74.8%; 95% CI 73.7, 75.8) with any lipid anomalies. However, adolescents living in rural settlements have higher proportions (76.8%; 95% CI 76.0, 77.6) of individuals suffering from any lipid anomalies than those living in urban areas (75.9%; 95% CI 74.4, 77.3).

Adolescents from poor and middle-class families have a significantly higher proportion of individuals suffering from any lipid anomalies with 77.9 (95% CI 77.5, 80.6), and 79.1 (95% CI 77.5, 80.6), respectively. When the caste of the adolescents was looked upon, adolescents belonging to the other category had the highest proportions (78.4%; 95% CI 77.0, 79.8) suffering from any lipid anomalies, followed by those belonging to other backward classes (76.7%; 95% CI 75.6, 77.9) and SC/STs (74.9%; 95% CI 73.5, 76.1). Adolescents from the western region of the country have the highest burden of any lipid anomalies (80.8%; 95% CI 79.1, 82.4), followed by those living in eastern India (78.2%; 95% CI 76.8, 79.6) and the north-eastern region (77.0%; 95% CI 74.7, 79.1). Muslim adolescents bear the highest burden, with approximately 80.4 percent (95% CI 78.6, 82.2) suffering from any lipid anomalies, followed by those from other religions (79.8%; 95% CI 76.4, 82.9). Lastly, 75.7 percent (95% CI 74.9, 76.5) of Hindus suffered from any lipid anomalies.

In addition, roughly 89.1 percent (95% CI 85.2, 92.0) of overweight or obese adolescents can be seen suffering from any lipid anomalies compared to those with under or normal-weight adolescents (i.e., 76.1%; 95% CI 75.4, 76.9). Individuals with vitamin A, D, and B12 deficiencies have 70.1 (95% CI 67.6, 72.5), 80.7 (95% CI 79.2, 82.1), and 73.4 (95% CI 71.9, 74.9) percent of individuals suffering from any lipid anomalies, respectively. Further, in those suffering from pre-diabetes, approximately 80.6 percent (95% CI 78.1, 82.9) suffer from any lipid anomalies compared to those with normal blood glucose levels (76.6%; 95% CI 75.7, 77.4). At the same time, those with zinc, folate, and iron-deficient adolescents have 76.7 (95% CI 75.7, 77.4), 75.6 (95% CI 74.3, 76.9), and 74.9 (95% CI 73.0, 76.7) percent of adolescents suffering from any lipid anomalies, respectively. Furthermore, those who consumed iron-folic and multivitamin supplements have 78.2 (95% CI 75.7, 80.5) and 77.6 (95% CI 75.1, 80.0) percent of adolescents suffering from any lipid anomalies, respectively.

Table 3 represents the prevalence of types of lipid profiles by the age of the adolescents categorized into two categories, i.e., early (10–14 years), late adolescents (15–19 years), and overall (10–19 years) in India.

**Table 3:** Prevalence of types of lipid profiles in early adolescence (i.e., 10–14 years) and late adolescence (15–19 years) in India, CNNS, 2016–18

Variables	Weighted prevalence (95% CI)	
	Early adolescence	Late adolescence
Hypercholesterolemia	54.61 (41.74, 53.49)	45.67 (41.24, 50.17)
Hypertriglyceridemia	53.86 (51.71, 55.99)	46.14 (44.01, 48.28)
Low-HDL-cholesterol (HDL-C)	48.27 (46.65, 49.89)	51.73 (50.11, 53.35)
High total cholesterol (TC): HDL-C ratio	43.49 (39.02, 48.06)	56.51 (51.94, 60.98)
Isolated hypercholesterolemia	56.94 (51.54, 61.24)	43.06 (38.76, 47.46)
Isolated hypertriglyceridemia	52.73 (51.43, 54.02)	47.27 (45.98, 48.57)
Isolated low HDL-C	52.50 (51.60, 53.41)	47.50 (46.59, 48.40)

Hypercholesterolemia is more prevalent among early adolescents with a prevalence of 54.61 (95% CI 41.74, 53.49) than late adolescents with 45.67 (95% CI 41.24, 50.17). Similarly, hypertriglyceridemia has a prevalence of approximately 53.86 (95% CI 51.71, 55.99) among early adolescents and 46.14 (95% CI 44.01, 48.28) among late adolescents. However, prevalence of low HDL-C can be seen as 48.27 (95% CI 46.65, 49.89) and 51.73 (95% CI 50.11, 53.35) can be seen among early and late adolescents, respectively. High total cholesterol to HDL-C ratio can be seen having a prevalence of 43.49 (95% CI 39.02, 48.06) among early adolescents and 56.51 (95% CI 51.94, 60.98) among late adolescents.

Further, isolated hypercholesterolemia has a prevalence of 56.94 (95% CI 51.54, 61.24) and 43.06 (95% CI 38.76, 47.46) can be seen among early and late adolescents, respectively. However, isolated hypertriglyceridemia has a prevalence with 52.73 (95% CI 51.43, 54.02) and 47.27 (95% CI 45.98, 48.57) among early and late adolescents, respectively. Lastly, isolated low HDL-C has a prevalence of 52.50 (95% CI 51.60, 53.41) and 47.50 (95% CI 46.59, 48.40) can be seen among early and late adolescents, respectively.

Table 4 shows the mean value of lipid profiles by total, early, and late adolescents in India (2016–18). Mean total cholesterol among early, late, and all adolescents were computed as 141.0 (SD = 32.3). 140.1 (SD = 33.5) and 140.6 (SD = 32.9) mg/dl, respectively. Similarly, mean triglycerides were calculated to be 95.3 (SD = 50.0) mg/dl whereas, mean triglycerides of 96.0 (SD = 49.7) and 94.5 (SD = 50.3) mg/dl among early and late adolescents, respectively. Further, the mean HDL-C was computed to be 47.3 (SD = 10.7) mg/dl where, mean HDL-C was calculated to be 48.2 (SD = 10.9) and 46.3 (SD = 10.7) mg/dl among early and late adolescents, respectively. Mean LDL-C among adolescents was computed to be 84.1 (SD = 24.8) mg/dl with 83.4 (SD = 23.9) and 84.9 (SD = 25.8) mg/dl among early and late adolescents, respectively. Lastly, the mean total cholesterol to HDL-C ratio was computed to be 3.05 (SD = 0.7) mg/dl. However, a mean of 3.0 (SD = 0.7) and 3.1 (SD = 0.8) mg/dl was calculated among early and late adolescents, respectively.

**Table 4:** Mean value of lipid profiles in late adolescence (i.e., 10–14 years) and early adolescence (15–19 years) and overall (10–19 years) in India, CNNS, 2016–18

Variables	Early adolescence	Late adolescence	Overall	t-test
	Mean (SD)			p-value
Total cholesterol	141.02 (32.32)	140.13 (33.54)	140.61 (32.89)	< 0.001
Triglycerides	96.03 (49.73)	94.49 (50.33)	95.31 (50.01)	< 0.001
HDL-C	48.15 (10.85)	46.29 (10.47)	47.29 (10.72)	< 0.001
LDL-C	83.43 (23.85)	84.86 (25.77)	84.09 (24.77)	< 0.001
Total Cholesterol: HDL-C ratio	3.01 (0.72)	3.11 (0.78)	03.05 (00.75)	< 0.001

**Fig. 1:**
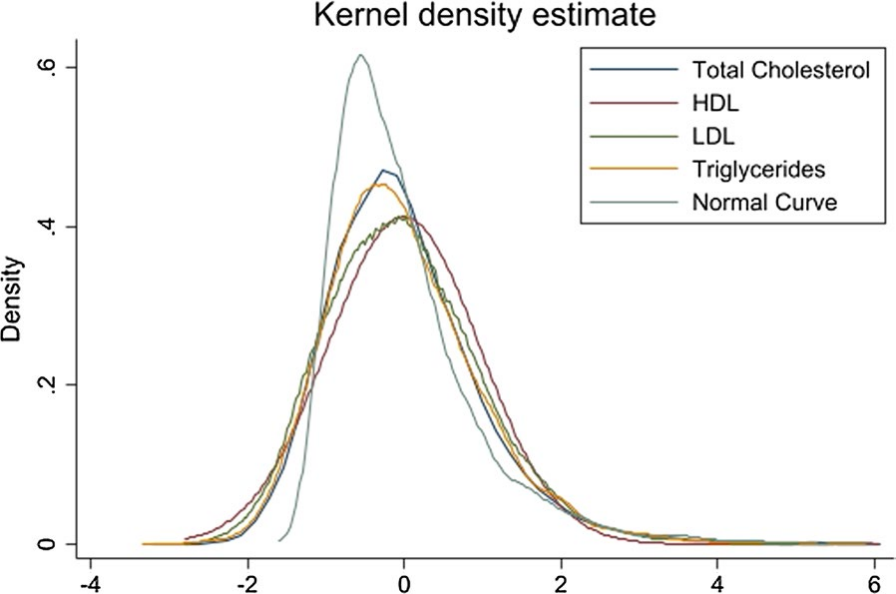
Kernel density curve for lipid profiles among adolescents in India. kernel = epanechnikov, bandwidth = 0.1216

Figure 1 depicts that standardized lipid profiles almost follow a normal distribution with mean “0” and variance “1”, as all the four curves lie near the standard normal curve.

**Fig. 2:**
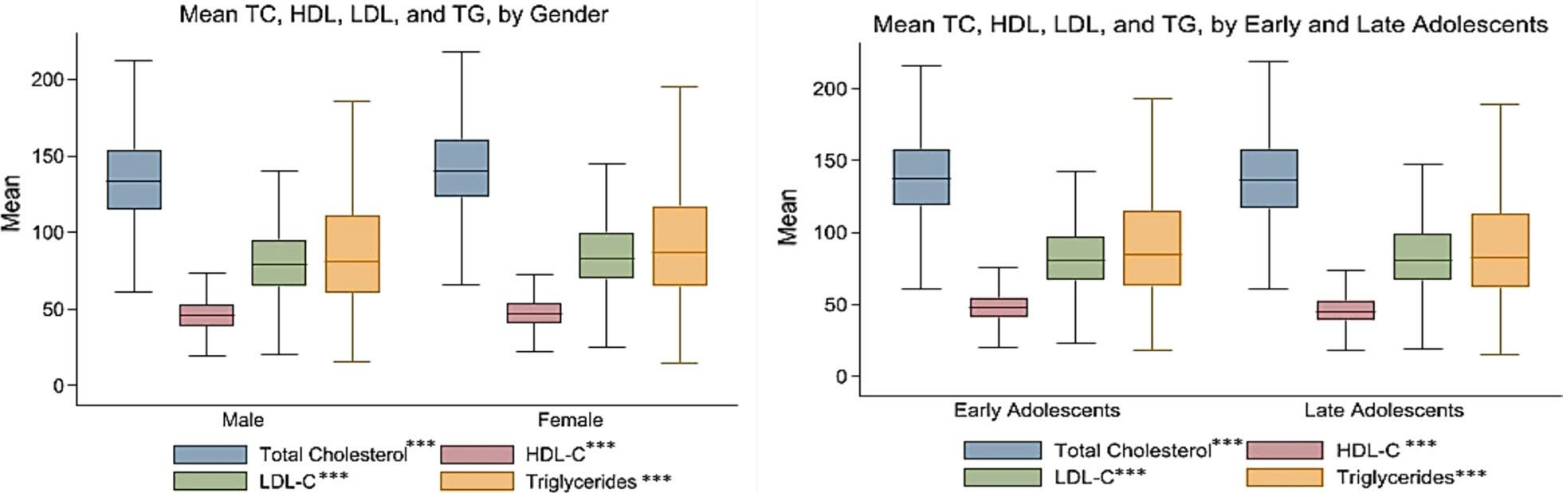
Box plot for lipid profiles by gender and age among adolescents in India. ***p-value for t-test < *0.001*

Figure 2 is a boxplot representing each lipid profile by gender and age (i.e., early and late adolescents). Using a t-test for the difference of mean for each lipid profile, mean values differ significantly when a comparison is made between males and females. Similarly, in early and late adolescents, there is a significant difference between the means of each lipid profile.

**Fig. 3:**
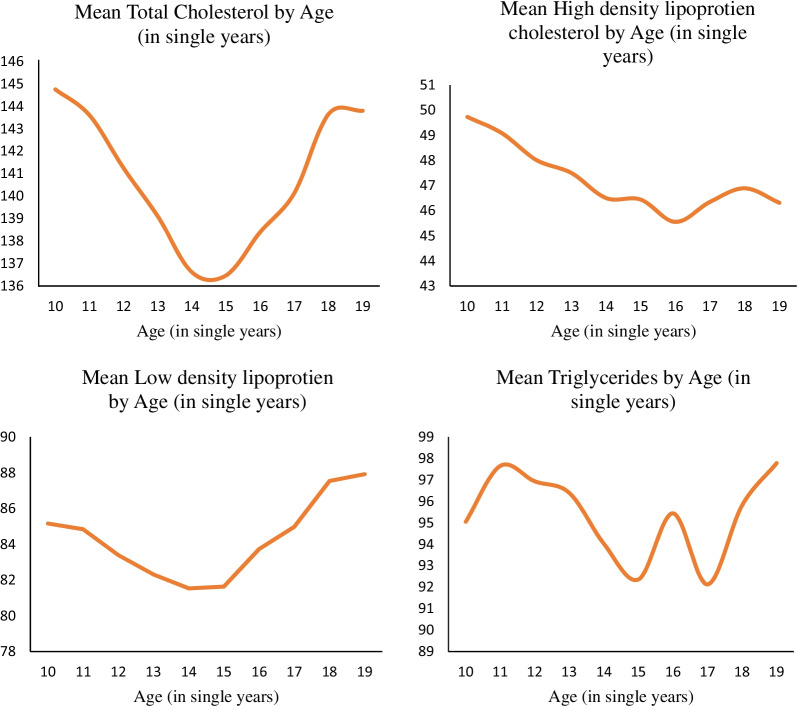
Distribution of each lipid profile by age of the adolescents in India

Figure 3 represents the distribution of each lipid profile by the age of the adolescents aged 10–19 years. A change in pattern can be seen in each of the mean lipid profiles by age in single years after attaining the age of fourteen to fifteen years. Mean total cholesterol can be seen following a declining pattern from the age of ten till the age of fourteen years. Then, the mean increases monotonically after attaining the age of fifteen among adolescents. Similarly, mean HDL-C among adolescents follows a declining pattern until age sixteen. At the same time, mean LDL-C is monotonically increasing significantly after the age of fourteen years after following a declining pattern until the age of fourteen among adolescents. However, no pattern is found in mean triglycerides among adolescents.

Table 5 represents the prevalence of any lipid anomalies by various micronutrient deficiencies. Approximately 70.1 (95% CI 67.6, 72.5) percent of the respondents who were vitamin A deficient suffered from any lipid anomalies. Similarly, 73.4 (95% CI 71.9, 74.9) and 80.7 (95% CI 79.2, 8.1) percent of the respondents with vitamin B12 and D deficiencies suffered from any lipid anomalies, respectively. However, among respondents with iron, zinc, and folate deficiencies there were 74.9 (95% CI 73.0, 77.0), 76.7 (95% CI 75.2, 78.1), and 75.6 (95% CI 74.3, 76.9) percent were suffering from any lipid anomalies.

**Table 5:** Prevalence of types of lipid profiles by micronutrient deficiencies among adolescents aged 10–19 years in India, CNNS, 2016–18 with 95% CI

Micronutrients deficiencies	No lipid anomalies	Any lipid anomalies
Vitamin A deficiencies	29.91 (27.51, 32.41)	70.09 (67.59, 72.49)
Vitamin B12 deficiencies	26.59 (25.13, 28.10)	73.41 (71.90, 74.87)
Vitamin D deficiencies	19.34 (17.93, 20.84)	80.66 (79.16, 8.07)
Iron deficiencies	25.11 (23.34, 26.97)	74.89 (73.03, 76.98)
Zinc deficiencies	23.31 (21.89, 24.79)	76.69 (75.21, 78.11)
Folate deficiencies	24.38 (23.10, 25.72)	75.62 (74.28, 76.90)

Table 6 depicts the proportions of adolescents aged 10–19 years suffering from no and any lipid anomalies by their frequency of consumption of unhealthy foods such as consumption of fats and oils, sugar, and jaggery, fried, junk foods, sweets, and aerated drinks; categorized into never, occasionally, and frequently consuming in a week. In adolescents who consumed fats/oils, sugar/jaggery, and fried foods frequently, approximately 75.9 (95% CI 74.91, 76.93), 76.7 (95% CI 75.82, 77.54), and 76.9 (95% CI 74.38, 79.21) percent were suffering from any lipid anomalies, respectively. Similarly, for those who consumed junk foods frequently, i.e., more than three days a week, roughly 73.7 (95% CI 68.33, 78.41) percent were suffering from any lipid anomalies. Further, among respondents who consume sweets and aerated drinks more often, i.e., more than three days a week, around 77.5 (95% CI 73.63, 80.93) and 77.6 (95% CI 73.55, 81.15) percent were suffering from any lipid anomalies, respectively.

**Table 6:** Proportions of adolescents (aged 10–19 years) suffering from no lipid anomalies and any lipid anomalies across the frequency of consumption of unhealthy diet in India (2016–18)

Unhealthy dietary habits	No lipid anomalies	Any lipid anomalies
**Fats and oils**		
Never	18.83 (16.60, 21.28)	81.17 (78.25, 83.40)
Occasionally	23.56 (22.41, 24.75)	76.44 (75.25, 77.59)
Frequently	24.07 (23.07, 25.09)	75.93 (74.91, 76.93)
**Sugar and Jaggery**		
Never	28.21 (22.61, 34.56)	71.79 (65.44, 77.39)
Occasionally	23.48 (22.11, 24.92)	76.52 (75.08, 77.89)
Frequently	23.31 (22.46, 24.18)	76.69 (75.82, 77.54)
**Fried foods**		
Never	20.70 (17.14, 24.77)	79.30 (75.23, 82.86)
Occasionally	23.57 (22.80, 24.36)	76.43 (75.64, 77.20)
Frequently	23.12 (20.79, 25.62)	76.88 (74.38, 79.21)
**Junk foods**		
Never	20.38 (19.06, 21.76)	79.62 (78.24, 80.94)
Occasionally	24.48 (23.61, 25.36)	75.52 (74.64, 76.39)
Frequently	26.32 (21.59, 31.67)	73.68 (68.33, 78.41)
**Sweets**		
Never	20.19 (17.65, 23.00)	79.81 (77.00, 82.35)
Occasionally	23.72 (22.95, 24.50)	76.28 (75.50, 77.05)
Frequently	22.51 (19.07, 26.37)	77.49 (73.63, 80.93)
**Aerated drinks**		
Never	21.65 (19.67, 23.77)	78.35 (76.23, 80.33)
Occasionally	23.73 (22.95, 24.54)	76.27 (75.46, 77.05)
Frequently	22.42 (18.85, 26.45)	77.58 (73.55, 81.15)
Total	23.44 (22.72, 24.17)	76.56 (75.83, 77.28)

## Discussion

Present study depicts that over three-quarters of the adolescents in the study, aged 10 to 19 years exhibit abnormalities in at least one of the lipid markers. As individuals shift from early, i.e., 10–14 years, to late adolescents (i.e., 15–19 years) burden of any lipid anomalies increases. In India, female adolescents have been found to bear a higher burden of lipid abnormalities compared to their counterparts, similar results reported by other studies, which can be attributed to the overall gender disparity in dietary diversity among adolescents in India [23]. However, there was no significant difference between those living in a rural and urban place of residence, unlike other studies’ results [10].

When analyzing the burden of any lipid anomalies by wealth quintile of the adolescent’s household, those belonging to the middle-income families bear the highest burden of any lipid anomalies. The Western region of the country has the highest proportion of adolescents suffering from any lipid anomalies. Muslim adolescents can be seen holding a significant number of individuals suffering from any lipid anomalies. Similar findings can be seen reported in another similar study [15].

A higher proportion of adolescents suffered from any lipid anomalies among those who were overweight or obese and pre-diabetics compared to each of their counterparts, which has proven to be a significant covariate of obesity and diabetes by various other studies [10, 24, 25]. Furthermore, a considerable number of individuals with vitamin A, D, and B12 deficits, as well as zinc, folate, and iron deficits, can be seen suffering from any lipid anomalies.

Additionally, the mean hypercholesterolemia is high among early adolescents. However, late adolescents are double burdened by high mean non-HDL-C and low mean HDL-C. That is, twice as high as mean total cholesterol to HDL-C ratio can be observed among late adolescents than early adolescents.

Individuals who consume an unhealthy diet very often bear a higher burden of individuals suffering from any lipid anomalies than others. However, when further desegregation of the unhealthy diet index was done, regardless of how often a person is consuming unhealthy diet (such as, fats and oils, sugar and jaggery, fried and junk foods, and aerated drinks are consumed in a week), a sizeable proportion of adolescents suffer from lipid abnormalities. Although, consuming jaggery can be effective in overcoming iron deficiency in an individual without any prominent side effects [26], but in present study it has been considered as an unhealthy diet practice as the data was gathered in conjunction with sugar intake in the survey. Furthermore, Food and Safety Standards Authority of India (FSSAI) has initiated the model “Front of Packaging Label” (FOPL) of packaged food products to reduce the impact of unhealthy food products on health. Which has found to be the most efficient way of influencing consumers’ behaviour towards consuming unhealthy food products [27]. Items high in sugar, sodium, and saturated fat are identified by FOPL, which is essentially a nutrient profile model. These foods would be given a warning label so that consumer could efficiently distinguish between more and less healthy foods [28]. However, unhealthy food products must bear a warning label.

In India, food diversification is lacking among children and adolescents. However, various studies have reported similar results regarding food diversification among adolescents in India and how it affects their nutritional status [29–31]. Therefore, lack of dietary diversity coupled with unhealthy dietary habit accentuates serious health issues be it section of the society.

The present study hint towards the derailing health among children and adolescents in India, consistent with other studies [9, 32–35]. Hence, appropriate intervention is needed to prevent the burden of unwanted and adverse health state on the individual as well as the health system in India. Limitations would be first, the fact that study is based on the cross-sectional data; second, that the study only explores the prevalence and distribution across various sub-groups of the population. However, an in-depth analysis is required to explore the risk factors of lipid anomalies among children and adolescents in India.

## Conclusion

Findings from the study suggest that proper lipid profile monitoring is required among adolescents in India. The high prevalence of lipid anomalies in India, particularly among children and adolescents, calls for urgent lifestyle intervention strategies to prevent and control this significant cardiovascular risk factor. Promoting healthy eating habits at the mentioned ages should be a part of education and learnings among school and college-going children and adolescents. Further, service providers should monitor and educate patients with diabetes and hypertension to adopt a healthy lifestyle and make them aware of the adverse health effect of such abnormalities at later ages. Policymakers should focus on strengthening the early detection and prevention of lipid abnormalities in children and adolescents in India.

## Data Availability

The CNNS data can be accessed from the Population Council (Delhi, India) upon request. The report and survey tools are available in the public domain on the following website: https://www.popcouncil.org/uploads/pdfs/2019RH_CNNSreport.pdf and data can be obtained on request from cnns.pc@gmail.com.
